# Outcome reporting for heavy menstrual bleeding

**DOI:** 10.52054/FVVO.14.3.031

**Published:** 2022-09-30

**Authors:** T.J. Clark

I write this editorial on holiday from the Greek island of Kos after returning from a visit to an ancient healing temple (an Asclepieion) where Hippocrates, the father of modern medicine, was said to have received his medical training. His oath of medical ethics emphasises that we must “first do no harm”. As doctors and surgeons, I am sure we all claim to adhere to the tenets of the Hippocratic oath, but we need to face the unpalatable fact that we will be doing “harm” unknowingly because at times we will be recommending suboptimal treatments. Let us take the highly prevalent condition of heavy menstrual bleeding (HMB) that adversely affects the quality of life (QoL) of women and girls and has knock on economic impacts on both the use of health services but also wider societal productivity. I know that many of you reading this will be keen endoscopic surgeons but how do you know that your decision for recommending surgery is the correct one? Are there better non-surgical treatments? Are there better alternative surgical options?

The article by Cooper et al. (2022) published in this issue attempts to collect, evaluate and summarise the outcomes used to examine the effectiveness of treatments for HMB. The authorship team should be congratulated for a herculean effort (apologies I am still imbibing the Greek vibes on my vacation!) in trawling through the world literature, including observational series for the first time, in addition to outcome data used in randomised controlled trials (RCTs). They discovered the use of over 200 outcomes for this one common condition! Thus, it seems that it is a case of ‘pick your favoured outcome(s)’. The lack of a systematic approach to assessing outcomes, and as a consequence effectiveness of treatments, presents huge potential dangers to our patients. A lack of rationality in formulating clinical endpoints when designing studies can lead to unethical research waste (because data may be irrelevant and importantly cannot be aggregated with similar studies in systematic reviews). Moreover, ‘loading the dice’ i.e., selecting outcomes in a specific population to increase the likelihood of positive outcomes, incentivises researchers as it enhances the likelihood of getting published. Even more worryingly, commercial pharmaceutical and medical device industry studies, also seek positive effectiveness outcomes in order to get regulatory approval and start marketing their wares as quickly as possible. Arguably, the fallout of such approaches to outcome evaluation has had undesirable impacts in benign gynaecology in recent times over the use of vaginal mesh and ulipristal acetate to name but two examples of promising recent interventions (Middelkoop et al., 2022).

So, if we are to do the best for our patients we need to acquire and implement the best, valid evidence to inform them and support our clinical decisions. Core Outcome Sets (COS) are certainly ‘of the moment’ because as Cooper et al. (2022) state they: “ensure that studies of a condition all report the same, valid outcomes which will allow future data synthesis for development of clinical guidelines and will also prevent selective outcome reporting. Ultimately, this will mean that all studies which are conducted into a condition will produce results that are not only useful for interpretation of that trial but can also contribute to meta-analyses and the overall assessment of interventions. This will make results more valuable, more meaningful in comparisons and more likely to influence improvements in policy and practice.” The first step in developing COS is to systematically evaluate the world literature and Cooper et al. (2022) have done this for us in an exemplary fashion. Whilst we await with bated breath the final COS that Cooper’s international team are developing, it is great that Facts, Views and Vision readers can see the evidence underpinning this process.

There are many interesting observations and insights from the Cooper et al. (2022) review. Some measure of menstrual blood loss was perhaps not surprisingly the most popular choice of primary outcome. However, this was assessed in several different ways with pictorial blood loss assessment charts (PBLAC) being most often used, maybe reflecting the regulatory requirements of the US Food and Drug Administration Agency (FDA) rather than a representation of clinical utility and face validity. That said arguably more patient centric and useful QoL and patient reported outcome measures (PROMs) were often chosen as a primary outcome. However, QoL and PROMs were the 5th most popular primary outcome after ‘menstrual blood loss’, ‘amenorrhoea’, ‘treatment success’ and ‘satisfaction’.

Until I read the review by Cooper et al. (2022), I was unaware that authors evaluating surgical interventions for HMB prefer amenorrhoea as a primary endpoint whereas authors assessing medical interventions preferentially chose some form of measured blood loss, although as discussed above, how such an outcome is measured lacks any consistency. Further, taking the apparently simple concept of ‘amenorrhea’ i.e., absence of menstrual bleeding, the Cooper et al. (2022) review identified eight different ways of assessing and reporting this endpoint! Whilst we are on the subject of amenorrhoea, why is this seemingly the holy grail of surgical outcome for HMB? Surely these biases towards total hysterectomy? Where does morbidity and recovery figure as an outcome when comparing surgical interventions or surgery with medical treatment? Adverse effects of treatments need be accounted for. In the case of global endometrial ablation for example, amenorrhoea rates of 40-50% can be expected and satisfaction rates in the order of 90% at one year (Clark et al., 2011). However, we know that hysterectomy rates at two years may be as high as 18% primarily because of ongoing, worsening, or new cyclical pelvic pain (Oderkerk et al., 2022). Thus, collection of data about side-effects and re-interventions is relevant as highlighted in this review and also decisions about when they should be measured.

As a clinical academic who has conducted both medical and surgical research into HMB, I have often thought when designing studies that a simple, global measure applicable across all types of interventions, i.e., non-medical, medical, radiological, and surgical, maybe best rather than relying on a plethora of quantified or semi-quantified measures, continuous or ordinal scales, and validated or unvalidated QoL tools. Cooper et al. (2022) found that overarching measures such as ‘satisfaction’ and ‘success’ were often reported but the scales used lacked consistency precluding valid comparison and data aggregation in meta- analyses. Moreover, it is unclear what such terms actually mean and refer to e.g., satisfaction with their care, satisfaction with the alleviation of their HMB or overall satisfaction with their health.

A final point I would like to raise is that treatment of HMB alone for many women may not be sufficient to impact positively on their overall QoL and physical functioning. HMB is defined by the United Kingdom National Institute for Health and Care Excellence as ”excessive menstrual blood loss which interferes with a woman’s physical, social, emotional and/or material quality of life and which can occur alone or in combination with other symptoms” (NICE, 2018). Thus, it is clear that related menstrual cyclical symptoms need to be taken into account to evaluate the impact of treatments not only on HMB but other associated cyclical symptoms such as pelvic pain, non-menstrual bleeding, and pre-menstrual physical / mental symptoms amongst others.

All research studies will have their own nuances and the need for specific outcome assessment and timings of assessments. However, a basic COS in HMB will be a great step forward in helping us to assess studies, compare or combine data from similar studies, compare with other treatments, generalise findings to our own patient populations and produce impactful clinical guidance. Hippocrates separated scientific truth from superstitions favoured by priests, physicians, and charlatans of the time. Almost 2500 years later, we must also strive to do the same; producing reliable and robust research to determine ‘scientific truth’. The use of meaningful clinical outcomes is of key importance to achieving this goal. The Cooper et al. (2022) review is an illuminating read and we look forward to the COS in HMB, based to a large degree on these data, being developed and disseminated in the near future.
